# Intranasal immunization with plasmid DNA encoding spike protein of SARS-coronavirus/polyethylenimine nanoparticles elicits antigen-specific humoral and cellular immune responses

**DOI:** 10.1186/1471-2172-11-65

**Published:** 2010-12-31

**Authors:** Byoung-Shik Shim, Sung-Moo Park, Ji-Shan Quan, Dhananjay Jere, Hyuk Chu, Man Ki Song, Dong Wook Kim, Yong-Suk Jang, Moon-Sik Yang, Seung Hyun Han, Yong-Ho Park, Chong-Su Cho, Cheol-Heui Yun

**Affiliations:** 1 Department of Agricultural Biotechnology and Research Institute for Agriculture and Life Sciences, Seoul National University, Seoul 151-921, Republic of Korea; 2 Department of Microbiology, College of Veterinary medicine, Seoul National University, Seoul 151-921, Republic of Korea; 3 Laboratory Science Division, International Vaccine Institute, Seoul 151-818, Republic of Korea; 4 College of Pharmacy, Yanbian University, Jilin Province 133000, PR China; 5 Division of Zoonoses, Center for Immunology & Pathology, National Institute of Health, Korea Center for Disease Control & Prevention, Seoul 122-701, Republic of Korea; 6 Division of Biological Sciences and The Institute for Molecular Biology and Genetics, Chonbuk National University, Jeonju 561-756, Republic of Korea; 7 Department of Oral Microbiology & Immunology, Dental Research Institute, and BK21 Program, School of Dentistry, Seoul National University, Seoul 110-749, Republic of Korea; 8 Center for Agricultural Biomaterials, Seoul National University, Seoul 151-921, Republic of Korea

## Abstract

**Background:**

Immunization with the spike protein (S) of severe acute respiratory syndrome (SARS)-coronavirus (CoV) in mice is known to produce neutralizing antibodies and to prevent the infection caused by SARS-CoV. Polyethylenimine 25K (PEI) is a cationic polymer which effectively delivers the plasmid DNA.

**Results:**

In the present study, the immune responses of BALB/c mice immunized via intranasal (i.n.) route with SARS DNA vaccine (pci-S) in a PEI/pci-S complex form have been examined. The size of the PEI/pci-S nanoparticles appeared to be around 194.7 ± 99.3 nm, and the expression of the S mRNA and protein was confirmed *in vitro*. The mice immunized with i.n. PEI/pci-S nanoparticles produced significantly (*P *< 0.05) higher S-specific IgG1 in the sera and mucosal secretory IgA in the lung wash than those in mice treated with pci-S alone. Compared to those in mice challenged with pci-S alone, the number of B220^+ ^cells found in PEI/pci-S vaccinated mice was elevated. Co-stimulatory molecules (CD80 and CD86) and class II major histocompatibility complex molecules (I-A^d^) were increased on CD11c^+ ^dendritic cells in cervical lymph node from the mice after PEI/pci-S vaccination. The percentage of IFN-γ-, TNF-α- and IL-2-producing cells were higher in PEI/pci-S vaccinated mice than in control mice.

**Conclusion:**

These results showed that intranasal immunization with PEI/pci-S nanoparticles induce antigen specific humoral and cellular immune responses.

## Background

Severe acute respiratory syndrome (SARS) is an emerging infectious disease [1]. In contrast to most other coronaviruses, which cause mild infection, the new SARS-CoV has a high mortality rate. Because the re-emergence of SARS is possible due to existence of SARS-CoV like strains in animal reservoir, development of safe and effective vaccines is highly desired. The SARS-CoV genome is composed of single positive stranded RNA and encodes four main structural proteins including spike protein (S), membrane protein (M), envelope protein (E) and nucleocapsid protein (N) [2]. The S protein is involved in not only receptor recognition but also in virus attachment and its entry into target cells [3].

In attempts to develop vaccines against various pathogens, research on DNA vaccine has been widely carried out. Using DNA vaccines, both humoral and cellular immune responses are induced [4]. A few studies demonstrated that DNA-based vaccines can induce protective immune response against several viruses [5,6]. However, one of the problems with DNA-based vaccines is that they are incapable of inducing immune response in mice when injected through the intranasal (i.n.) route [7]. In light of the fact that the entry of most respiratory diseases is through the mucosal surface, it is obvious and ideal that a vaccine should induce both systemic and mucosal immune responses. Secretory IgA plays a major role in mediating mucosal immunity [8]. Mucosal immune responses take an important role as a first line of immune defense system against influenza virus infection although parenteral immunization is not enough to provoke protective immunity [9].

Polyethylenimine (PEI) has been widely used as the non-viral vector *in vitro *and *in vivo *due to high transfection efficiency and buffering capacity [10]. It has been shown that mucosal administration with PEI could function as a potent mucosal immunostimulator [11]. It has been revealed that PEI is a very effective gene delivery vehicle for lung transfection producing high antibody titers against the encoded protein [12]. In the present study, the immune responses in BALB/c mice immunized with SARS DNA vaccine via i.n. route have been examined.

## Results

### Characterization of PEI/pci-S complexes

**Figure 1:**
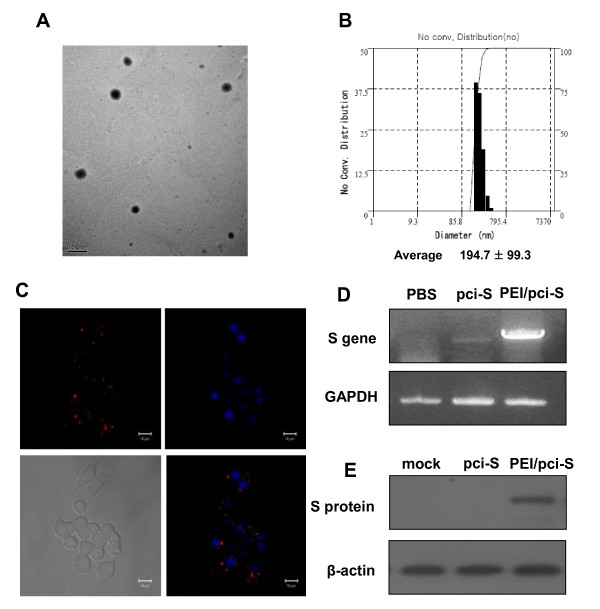
**Characterization of SARS-CoV S DNA vaccine (pci-S)-PEI complexes**. (A) Transmission electron micrographic image of PEI/pci-S complexes at N/P ratio 10. Scale bar represents 0.5 μm. (B) Size distribution of complexes prepared at N/P ratio 10. (C) The cell uptake of PEI/pci-S complexes was observed by confocal laser scanning microscope. Upper left, intracellular distribution of rhodamine-labeled PEI/pci-S (red). Upper right, cell nuclei by DAPI staining. Lower left, differential interference images of RAW 264.7 cells. Lower right, overlapping image of nuclei and rhodamine-labeled PEI/pci-S. (D) Expression of S mRNA in RAW 264.7 cells transfected with PEI/pci-S complexes was detected by RT-PCR. (E) S protein in RAW 264.7 cells with PEI/pci-S complexes was detected by Western blot.

It is well known that transfection efficiency of gene carrier depends upon its ability to condense DNA into nano-sized particles [13]. As expected, PEI condensed DNA into nano-sized particles, suggesting their endocytosis potential (Figure 1A).

The formation of PEI/pci-S nanoparticles was further confirmed by morphology observation. Representative energy-filtering transmission electron microscopy (EF-TEM) images of the PEI/pci-S nanoparticles at N/P ratio 10 are shown in Figure 1B. The nanoparticles were observed as spherical shape with around 200 nm size, which are similar to those measured by dynamic light scattering. It is notable that cytotoxicity of PEI was measured in RAW 264.7 cells after transfection with PEI/pci-S complexes by using MTT assay. The cell viabilities decreased slightly when the N/P ratios of PEI/pci-S complexes increased. When the N/P ratio was 10, the cell viability was 87.5 ± 7.3% (data not shown). In order to confirm PEI/pci-S uptaken by RAW264.7 cells, rhodamine labeled pci-S DNA was used to form the nanoparticles with the PEI and the complex was visualized by confocal microscopy. As shown in Figure 1C, the labeled nanoparticles can be seen in the cells, near to the nucleus. RT-PCR analysis showed both pci-S DNA and PEI/pci-S nanoparticles can transfect the cells. In fact, the PEI/pci-S nanoparticles induced much stronger S mRNA expression than that of naked DNA (Figure 1D). Western blot analysis further confirmed that the cells treated with PEI/pci-S nanoparticles can induce the expression of detectable S protein whereas no protein was detected when pci-S was used alone (Figure 1E).

### SARS-CoV S-specific antibody responses and B cell proliferation capacity

**Figure 2:**
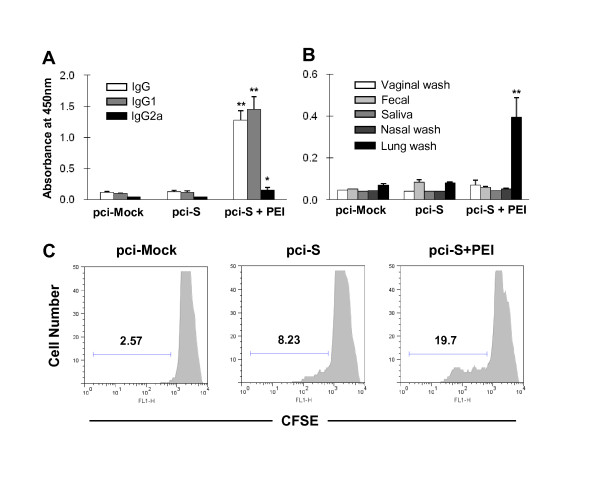
**SARS-CoV S protein-specific humoral and mucosal immune responses in BALB/c mice intranasally immunized with PEI/pci-S complexes**. The samples were collected on day 7 after the last immunization. Induction of (A) S-specific IgG subclasses in serum and (B) S-specific IgA in various mucosal samples were determined by ELISA. The results were expressed as means ± SEM for the group (n = 5 to 8). Significant differences compared with pci-S group were expressed as *P < 0.05 and **P < 0.01, respectively. (C) Proliferation activity of B220^+ ^cells from spleen of mice immunized with PEI/pci-S complexes. Bar and number in each panel present the percentage of proliferated cells.

To evaluate the influence of PEI on adaptive immunity to SARS-CoV S protein, specific antibody responses were examined in mice immunized i.n. with SARS-CoV DNA vaccine. Immunization with PEI/pci-S complexes elicited high level of SARS-CoV S-specific-serum IgG antibody but not in mice immunized with SARS-CoV S DNA alone (Figure 2A). To access the balance of Th1/Th2 response, SARS S-specific IgG1 and IgG2 were evaluated in immunized mice. SARS S-specific IgG1 antibody was significantly (*P *< 0.01) increased in mice immunized with SARS-CoV S DNA vaccine plus PEI whereas little increase was observed on SARS-specific IgG2a antibody production (Figure 2A), indicating Th2 dominant response. To examine mucosal antibody production, lung wash, nasal wash, fecal extracts, saliva and vaginal wash of immunized mice were collected. The result showed that SARS S-specific IgA antibody response was significantly (*P *< 0.01) increased in lung wash from mice immunized with PEI/pci-S complexes (Figure 2B).

To assess B lymphocyte proliferation against SARS-CoV spike protein, the notion that antibody responses enhanced was further confirmed by proliferation ability of B220^+ ^cells at 1 week after the last vaccination. B220^+ ^cells from mice immunized with PEI/pci-S complexes were highly proliferated after *in vitro *re-stimulation with SARS-CoV S protein (Figure 2C).

### Expression of cell surface molecules and maturation of dendritic cells (DCs) from the mice stimulated with PEI/pci-S complexes

**Figure 3:**
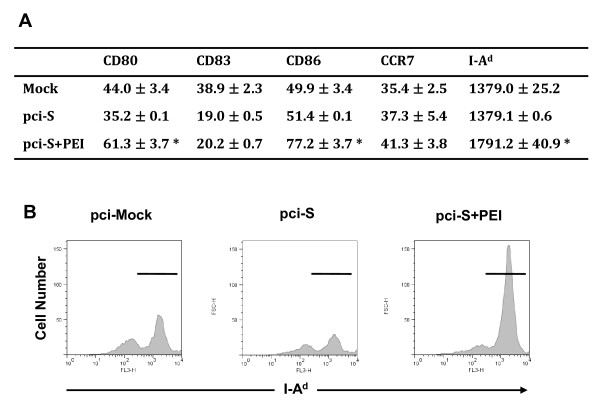
**Expression of cell surface molecules, CD80, CD83, CD86, CCR7 and MHC class II, on CD11c^+ ^cells in cervical lymph nodes from mice immunized with PEI/pci-S complexes**. BALB/c mice were immunized with PBS, pci-mock, pci-S, or PEI/pci-S complexes and then cell surface molecules on CD11c^+ ^cells from cervical lymph nodes were analyzed on day 3 after the last immunization. (A) The expressions of major cell surface molecules, CD80, CD83, CD86, CCR7, and MHC class II (I-A^d^) on CD11c^+ ^DC were determined by flow cytometry. Data were expressed as the mean value of mean fluorescence intensity ± SD. Significant differences compared with pci-S group were expressed as *P < 0.05. (B) Expression of MHC class II molecules was represented by histogram.

The maturation of DCs is accompanied with enhanced expression of surface markers, including co-stimulatory and MHC class molecules. To examine the effect of DNA vaccination on DC maturation *in vivo*, mice were immunized i.n. with PEI/pci-S complexes. The surface expression of CD80 and CD86 co-stimulatory molecules were significantly (*P *< 0.05) higher on DCs from mice treated with PEI/pci-S complexes than those from SARS-CoV DNA S vaccine alone (Figure 3). MHC class II, I-A^d^, expression was also up-regulated significantly (*P *< 0.05) in PEI/pci-S complexes group as compared with that of SARS-CoV DNA alone (Figure 3).

### Improvement of SARS-CoV S-specific T cell responses by PEI/pci-S complexes

**Figure 4:**
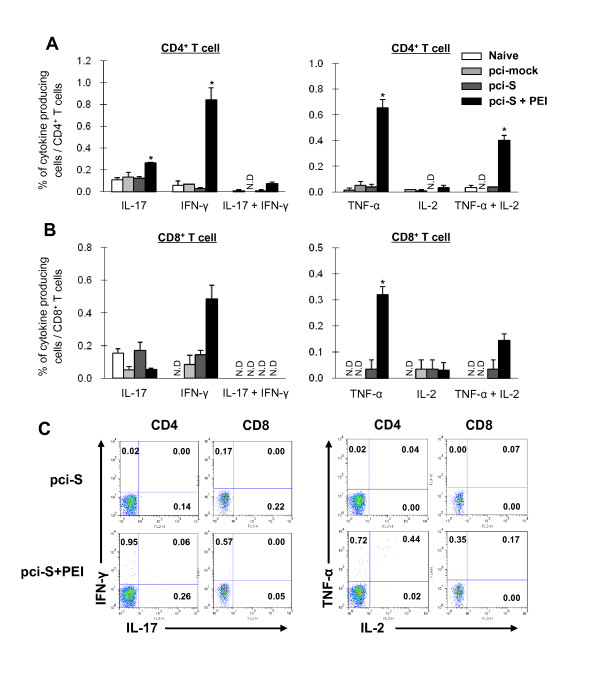
**Effector CD4^+ ^and CD8^+ ^T cell responses in BALB/c mice immunized with PEI/pci-S complexes**. Multi-intracellular cytokine staining for IL-17, IFN-γ, TNF-α and IL-2 was performed on (A) CD4^+ ^and (B) CD8^+ ^T cells after *in vitro *re-stimulation with SARS peptide. SARS S-specific CD4^+ ^T cells from lung were recovered on day 6 after the last immunization. ND indicates not detectable. Data were expressed as the mean value of mean fluorescence intensity ± SEM. Significant differences compared with pci-S group were expressed as *P < 0.01. (C) The results showed representative example of flow cytometry analysis.

To examine T cell immunity to SARS-CoV S DNA vaccine, cytokine profiles were examined by using intracellular cytokine assays. The cells were harvested from the lung at 6 days after the immunization. It has been suggested that T cells producing IFN-γ, IL-2, IL-17, and TNF-α are especially effective in protective immunity [14]. Amount of IFN-γ-producing cells were increased in CD4^+ ^and CD8^+ ^T cells from mice immunized with PEI/pci-S whereas IL-17-producing cells were increased only in CD4^+^, not CD8^+ ^T cells. It is notable that IFN-γ^+^, IL-2^+ ^and IL-17^+ ^cells were not detected in CD8^+ ^T cells from the mice immunized with PEI/pci-S complexes (Figure 4A and 4B). Re-stimulation with SARS-CoV S peptide induced the activation of cytokine-producing CD4^+ ^and CD8^+ ^T cells with a predominant production of TNF-α as well as TNF-α and IL-2 double cytokine-producing T cells in mice immunized with PEI/pci-S complexes (Figure 4A and 4B). Furthermore, IFN-γ and IL-17 double cytokine-producing cells were found more in the PEI/pci-S complexes group while it was not detectable in pci-S group. It is to note that IL-4-producing cells were detectable neither in the lung nor spleen (data not shown).

## Discussion

In the twenty first century, SARS was the first emerging infectious disease that has seriously threatened public health and the economy throughout the world [1]. Over 8,000 people from 26 countries were infected with SARS-coronavirus, resulting 774 deaths [15]. It has been shown that SARS-CoV spike protein (S) plays an important role in receptor recognition, virus attachment and its entry [16]. It represented one of the most important targets for the development of SARS vaccine [6]. To prevent and control SARS outbreaks, several vaccine studies based on the spike protein of SARS have been done including S protein vaccine, fragment DNA vaccine, full-length DNA vaccines and receptor binding domain [17]. DNA vaccine encoding full-length S protein has shown to induce humoral, cellular and protective immune responses against SARS-CoV [6]. In the current study, we evaluated the immunogenicity of a PEI/pci-S in mice through intranasal immunization.

There have been several reports that PEI/DNA complexes enhance transfection efficiency in mammalian cells and augment immunogenicity [18]. In the present study, we have adapted this PEI/pci-S complex for mucosal DNA vaccination. The size of PEI/pci-S complexes appeared to be about 200 nm. The effect of PEI/DNA complexes on transfection, and gene and protein expression in RAW 264.7 cells were evaluated by measuring the expression of the mRNA and protein, respectively. This result, thus, let us move ahead to *in vivo *studies for mouse immunization via intranasal route using PEI/pci-S complexes.

A number of studies attempted to develop SARS DNA vaccines, exclusively via systemic routes including intramuscular injection [19]. As SARS is a respiratory pathogen, among the SARS vaccine candidates, targeting intranasal immunization was likely to be more effective to induce protective immune responses against infection when compared with other delivery routes. Intranasal immunization of PEI/pci-S complexes induced higher antigen-specific serum IgG responses than pci-S alone. Coincidently, antigen-specific IgG1 was also dramatically increased when compared to IgG2a, suggesting Th2 dominant response. Also the immunization enhanced antigen-specific IgA response in bronchoalveolar lavage fluid and B cell proliferation after *in vitro *re-stimulation with the spike protein. In Garzon's study, antigen-specific antibody and T cell responses have been dose-dependently increased in mice immunized with DNA vaccine up to 100 μg [20]. However, in our study each mouse was immunized with only 20 μg of DNA. Despite the small amount of DNA, it has induced not only systemic but also mucosal immune responses.

DCs play a pivotal role for the effective induction of antigen-specific immune responses. DCs are found throughout the body and are considered as one of the first-line sentinel cells [21]. When DCs recognize pathogen-associated molecular patterns from microorganisms, DCs became mature and acquired capacity for the antigen presentation, concomitantly augmented the expression of MHC proteins [22], cytokines [23] and number of co-stimulatory molecules including CD80, CD83, and CD86 [24]. Thus, the maturation of DCs is essential for appropriate initiation of the subsequent adaptive immune responses [22]. In the present study, we demonstrated that the PEI/pci-S complexes increase the co-stimulatory and MHC class II molecules on DCs from cervical lymph nodes after intranasal immunization.

The cellular immune responses are mediated by both CD4^+ ^and CD8^+ ^T cells. Functional study indicated that antigen-specific T cells produce cytokines including IFN-γ, TNF-α, IL-2, and IL-17 after *in vitro *re-stimulation with SARS spike peptides. IFN-γ is an effector cytokine, critical for activating macrophages and DCs and inhibiting viral infection [25]. TNF-α is a cytokine that probably regulates immune cells and inhibits viral replication [26]. IL-2 mediates the expansion of T cells and maintains memory T cells [27]. IL-17 mediates the production of antimicrobial peptide and immunoglobulin for neutralizing viral infection [28]. In the current study, we have shown that antigen-specific CD4^+ ^and CD8^+ ^T cells secreted IFN-γ, TNF-α, IL-2, and IL-17 in non-lymphoid tissues such as lung. Furthermore multiple-cytokine producing cells are increased in mice immunized i.n. with PEI/pci-S. It is probable that these cells are likely responsible for the protection when host is infected with SARS-CoV after the vaccination. In fact, it has been suggested that multi-cytokine producing antigen-specific CD4^+ ^T cells are functionally superior on protection to single-cytokine producing cells [29]. There are several reports that multi-cytokine producing T cells have shown to correlate with protection against *Leishmania major *infection [30]. In the current study, the PEI/pci-S complexes induced a high frequency of TNF-α^+ ^IL-2^+ ^CD4^+ ^T cells and IFN-γ^+ ^IL17^+ ^CD4^+ ^T cells, and TNF-α^+ ^IL-2^+ ^CD8^+ ^T cells when assessed by SARS peptide recall responses. Taken together, our results support the hypothesis that intranasal immunization with PEI/pci-S complexes induces ideal cellular responses for the protection.

## Conclusion

PEI is effective in delivering DNA onto the mucosal surface, in maturation of dendritic cells and in improving the immunogenicity of the DNA vaccine. Our results indicated that PEI can be used as a vector for the mucosal delivery of DNA vaccine and play an important role in B cell and T cell immunities.

## Methods

### Construction of plasmid expressing SARS-CoV S protein

The gene encoding SARS-CoV spike (S) protein without transmembrane domain (amino acids 14-1154) was synthesized. The sequence was codon optimized for mammalian cell expression and the natural signal sequence was replaced with the leader sequence of human tissue plasminogen activator (tPA). The tPA-S gene and pci-neo (Promega, Madison, WI) were digested with Nhe I and Not I. Then, the plasmid expressing SARS-CoV S protein was generated by ligation.

### Particle size and morphology of the PEI/pci-S nanoparticles

PEI/pci-S nanoparticles were prepared by mixing polymer and pci-S DNA in a solution form at N/P (PEI/pci-S) ratio of 10. The size of PEI/pci-S nanoparticles was measured by an electrophoretic light scattering spectrophotometer (ELS8000, Otsuka Electronice, Osaka, Japan). Morphology of the PEI/pci-S nanoparticles was observed by EF-TEM (LIBRA 120, Carl Zeiss, Germany).

### Cell uptake

For cell uptake observation, pci-S DNA was labeled with rhodamine by using Label IT^® ^Tracker™ CX-Rhodamine kit (Mirus, WI). RAW 264.7 cells were seeded in the plate. PEI/Rhodamine-labeled pci-S DNA nanoparticles were incubated for 1 h and washed. Then, they were mounted using ProLong^® ^Gold antifade reagent with DAPI (Invitrogen, Carlsbad, CA). The cell uptake images were observed by confocal laser scanning microscope (Carl Zeiss-LSM510, Thornwood, NY).

### Expression of SARS-CoV S gene and protein

The expression of SARS-CoV S was examined in RAW 264.7 cells at both transcriptional and protein level. The cells were transfected with naked pci-S DNA or PEI/pci-S nanoparticles at N/P ratio of 10. The cells were lysed with Trizol or cell lysis buffer [31].

Reverse transcription (RT) were performed using Superscript III reverse transcriptase (Invitrogen). The resulting cDNA was amplified by PCR. The sequence of primers were as following; for pci-S, forward (CGT CGT GAA AGG CGA TGA TG) and reverse (CGA TGG TGT TGT TGC TGT AGG); for glyceraldehyde 3-phosphate dehydrogenase (GAPDH), forward (ACCACAGTCCATGCCATCAC) and reverse (TCCACCACCCTGTTGCTGTA). The RT-PCR products were analyzed by electrophoresis.

For Western blot assay, equal amount of lysates was separated by SDS-PAGE and subsequently transferred onto a nitrocellulose membrane (Amersham Biosciences, Piscataway, NY). Membranes were blocked with 5% non-fat milk. Spike protein primary antibody received from Chiron and horseradish peroxidase-conjugated secondary antibody (Santa Cruz Biotechnology, Inc., Santa Cruz, CA) were incubated with the membrane, in turn. Antigen-antibody interaction was detected with an ECL fluorescence system. *β*-actin was used as a control.

### Immunization of mice

Six- to eight-week old female BALB/c mice (Orient, Korea) were anesthetized. Five mice per group were immunized i.n. with 20 μg of pci-mock, pci-S, or PEI/pci-S complexes in total of 25 μl ultrapure water on days 0, 14, 28, and 42. All studies were approved by IACUC at International Vaccine Institute (Seoul, Korea).

### Sample collection

Sera and mucosal samples were collected on day 6 or 7 after the last immunization. Blood was collected from the retro-orbital plexus. Fecal extracts were dissolved in phosphate-buffered saline (PBS) containing 0.02% sodium azide. For other samples, mice were anesthetized and vaginal washes were collected by pipetting with PBS. Lung washes were performed by repeated flushing and aspiration with PBS into the lungs. Nasal washes were collected twice by flushing with PBS through the nasal cavity.

### Enzyme-linked immunosorbent assay (ELISA)

Microtiter plates (Nunc, Denmark) were coated with 2 μg/ml of S protein (Protein Sciences Corporation, Meriden, CT). Plates were blocked with 5% skim milk. Serum (1:20) or mucosal samples (1:2) except lung wash (no dilution) were diluted in the blocking buffer. Each 100 μl samples were applied into separate wells. Goat-anti-mouse IgG, IgG1, IgG2a or IgA conjugated with horseradish peroxidase (Santa Cruz Biotechnology, Inc) in the blocking buffer was added to each well. Color was developed with TMB solution (Sigma) in dark. The reaction was stopped by 0.5 N HCl. The absorbance at 450 nm was measured in a microplate reader (Molecular Devices Corp., Menlo Park, CA).

### B cell proliferation

Mice were sacrificed on day 7 after the last vaccination and spleens were collected. Splenocytes were labeled with Carboxyfluorescein succinimidyl ester (CFSE) (Invitrogen) and stimulated with 2 μg/ml SARS-S protein for 5 days. B cells were stained with anti-B220-PerCP (BD Biosciences). The degree of proliferation was detected using flow cytometry, FACSCalibur (BD Biosciences). All cytometric data were analyzed by using FlowJo software (Tree Star, San Carlos, CA).

### DC maturation *in vivo*

Cervical lymph nodes (CLN) were removed on day 3 after the last vaccination. Single cell suspension was stained with following antibodies: CD11c-APC and CD80-PE, CD83-PE, CD86-biotin, or I-A^d^-biotin (BD Biosciences). The degree of expression was detected using flow cytometry, FACSCalibur. All cytometric data were expressed as MFI (mean fluorescence intensity) and analyzed by using FlowJo software.

### Intracellular cytokine staining

Lungs, removed from mice on day 6 after the last vaccination, were disrupted into single-cell suspensions. The cells were seeded onto 96-well plate at 2 × 10^5 ^cells per well and re-stimulated with SARS peptide (Peptron) at 5 μg/ml for 12 hrs. Intracellular cytokine staining assay was followed by the manufacturer's instruction. The cells were stained with anti-CD4-PerCP and anti-CD8-FITC together with anti-IFN-γ-APC and -IL-17-PE, or anti-TNF-α-APC and -IL-2-PE (all from BD Biosciences). The percentage of cells with associated fluorescence was determined by using a flow cytometry, FACSCalibur. All cytometric data were analyzed by using FlowJo software.

### Statistical analysis

Statistical tests were performed by using Student's *t *test. *P *value less than 0.05 was considered significant.

## Abbreviations

SARS: severe acute respiratory syndrome; CoV: coronavirus; PEI: polyethylenimine 25K; S: spike protein; M: membrane protein; E: envelope protein; N: nucleocapsid protein; i.n.: Intranasal; pci-S: SARS DNA Vaccine; Ag: antigen; DCs: Dendritic cells; PAMPs: pathogen-associated molecular patterns; tPA: tissue plasmnogen activator; EF-TEM: energy-filtering transmission electron microscopy; CLN: cervical lymph nodes

## Competing interests

The authors declare that they have no competing interests.

## Authors' contributions

BSS, SMP and JSQ performed most of experiments, analyzed data, and carried out some statistical analysis. They participated in the design of the study and helped with draft of the manuscript. DJ carried out the assay, measuring the size of PEI/pci-S nanoparticles. HC performed statistical analysis. MKS, DWK, YSJ, MSY, SHH and YHP advised with analysing the data and drafting the manuscript. CSC and CHY conceived and designed the study and finalized the manuscript. All authors have read and approved the final manuscript.
